# Clinical Features of Bipolar Disorder Patients with Metabolic Syndrome: A Cross Sectional Clinic Based Study

**DOI:** 10.1192/j.eurpsy.2022.430

**Published:** 2022-09-01

**Authors:** O. Sahmelikoglu Onur, Ö. Baş Uluyol

**Affiliations:** 1 Bakirkoy Research & Training Hospital for Psychiatry, Neurology and Neurosurgery, Psychiatry, Istanbul, Turkey; 2 aSancaktepe Şehit Prof. Dr. İlhan Varank Research & Training Hospital, Psychiatry, İstanbul, Turkey

**Keywords:** metabolic syndrome, bipolar disorder, diabetes

## Abstract

**Introduction:**

Metabolic Syndrome (MS) is highly prevalent in patients with bipolar disorder(BD), and may affect the functionality of this population.The increased rate of MS in BD might be due to poorer access to physical health care, unhealthy lifestyle related with psychiatric symptoms and adverse effects of pharmacological treatments.

**Objectives:**

We sought to compare differences in clinical features of patients with Bipolar Disorder Type 1 (BPD-1)with and without MS in euthymic period.

**Methods:**

This study included 67 euthymic BPD-1 patients without MS and 33 age- and sex-matched BPD-1 patients with MS. All participants completed a sociodemographic form; took the Beck Anxiety Inventory (BAI), Beck Depression Inventory (BDI) and Young Mania Rating Scale score. MS was diagnosed according to the International Diabetes Federation (IDF) criteria.

**Results:**

Age at onset of disease was significantly lower in BPD-1 group with MS than without MS (p < 0.05). Number of suicide attempts, psychiatric hospitalisation, was significantly higher in BPD-1 group with MS than without MS (p < 0.05). Catatonic and melancholic depression were significantly more prevalent in the BPD-1 with MS than without MS (p < 0.05). Having history of comorbid psychiatric diseases, mixed episodes, are significantly more prevalent in the BPD-1 with MS than without MS (p < 0.05). There was not significant difference between groups in terms of the medication between groups (p>0.05)

**Conclusions:**

Our findings suggest that MS might have an effect on the course of BD patients. The development and testing of interventions for preventing and treating the MS and its components in patients with BD might be important .

**Disclosure:**

No significant relationships.

